# A Phase Ib/II Study of Ganetespib With Doxorubicin in Advanced Solid Tumors Including Relapsed-Refractory Small Cell Lung Cancer

**DOI:** 10.3389/fonc.2018.00064

**Published:** 2018-03-12

**Authors:** Deepa S. Subramaniam, Stephen V. Liu, Jeanette Crawford, Jenna Kramer, Jillian Thompson, Hongkun Wang, Giuseppe Giaccone

**Affiliations:** ^1^MedStar Georgetown University Hospital, Washington, DC, United States; ^2^Georgetown University, Washington, DC, United States

**Keywords:** small cell lung cancer, ganetespib, doxorubicin, Hsp90 inhibition, chemotherapy resistance

## Abstract

**Introduction:**

Small cell lung cancer (SCLC) accounts for 15% of all lung cancers and is characterized by high response rates to cytotoxic chemotherapy and equally high rates of relapse. Many resistance mechanisms have been proposed including resistance to doxorubicin *via* induction of a heat shock response. Ganetespib is a novel and potent non-geldanamycin heat shock protein 90 (Hsp90) inhibitor. In preclinical studies, synergy between ganetespib and doxorubicin was shown. We conducted a phase Ib/II study of the safety, tolerability, and preliminary efficacy of the combination of ganetespib and doxorubicin.

**Methods:**

Patients eligible for the phase Ib portion had advanced tumors that would be appropriate for doxorubicin therapy and those in the phase II portion had relapsed or refractory SCLC. All patients had an ECOG performance status, 0–1 and adequate organ function, including a cardiac ejection fraction ≥50%. Patients who received a lifetime cumulative doxorubicin dose of >150 mg/m^2^ or who had symptomatic brain metastases were excluded. Patients received ganetespib on Days 1 and 8 and doxorubicin 50 mg/m^2^ on day 1 in 21-day cycles.

**Results:**

Eleven patients were enrolled including nine in the phase Ib dose escalation and two in the phase II expansion. The study was terminated by the sponsor. The dose recommended for future study is ganetespib 150 mg/m^2^ in combination with doxorubicin at a dose of 50 mg/m^2^. The most common adverse events of the combination were grade 1/2 diarrhea, nausea, fatigue, and transaminitis. No dose limiting toxicities were observed. Response rate was 25% and median duration of response was 137 days.

**Conclusion:**

Ganetespib plus doxorubicin was a well-tolerated combination and there remains potential for the clinical development of Hsp90 inhibitors in SCLC.

**Clinical Trial Registration:**

https://ClinicalTrials.gov/ct2/show/NCT02261805, identifier NCT02261805.

## Introduction

Small cell lung cancer (SCLC) accounts for about 15% of all lung cancers and is a leading cause of cancer-related mortality (1). This disease follows an aggressive course with median survival ranging from 12–20 months in patients with limited stage disease, with only 6–12% of patients living beyond 5 years. Nearly 60–70% of patients have extensive stage disease at initial presentation and <5% of patients with extensive stage disease live beyond 2 years. While objective response rates (ORRs) to first line combination chemotherapy, such as cyclophosphamide, doxorubicin, vincristine (CAV), cyclophosphamide, doxorubicin, etoposide, or cisplatin/carboplatin plus etoposide are robust at 60–70% in those with extensive stage disease, eventually resistance develops to chemotherapy and relapses typically occur early within 3–6 months (2).

Early trials of teniposide in the treatment of SCLC identified the influence of prior chemotherapy on response rates to subsequent treatment (3). Two major categories of relapse have been defined: platinum-sensitive relapse and platinum-resistant/platinum-refractory relapse. *Platinum-sensitive relapse* is frequently defined as relapse that occurs beyond 90 days of completion of platinum-based combination chemotherapy. In such patients, early trials of reinduction chemotherapy with the same regimen used at initial diagnosis produced an ORR of 50% (4). *Platinum-resistant relapse* is defined as relapse that occurs within the first 90 days of completion of platinum-based combination chemotherapy. Primary refractoriness, defined as tumor progression during treatment with platinum-based chemotherapy, carries a particularly poor prognosis. Relapsed/refractory SCLC (RR-SCLC) has a poor prognosis with median overall survival of only 2–3 months. Topotecan, a topoisomerase I inhibitor, is the only agent shown to improve overall survival compared to best supportive care in relapsed SCLC (5), and both oral and IV topotecan are currently approved in the United States in this setting. Amrubicin, another topoisomerase inhibitor approved in Japan for relapsed SCLC, was shown to be superior to topotecan in a study done in the Japanese population (6), but the results were not replicated in the Western population (7). Objective responses to single agent newer chemotherapy agents range from 14 to 29% (8).

Heat shock protein 90 belongs to a class of molecular chaperone proteins which help to regulate the folding, stability, and function of many Hsp90 client proteins. Hsp90 inhibition leads to conformational aberrations of the proteins, which are then targeted for ubiquitination and degradation by the proteasome (9, 10). Hsp90 clients include wild type or mutated forms of many oncoproteins associated with cancer, such as HIF-1α, hepatocyte growth factor receptor (cMET), and vascular endothelial growth factor receptor (11). Hsp90 inhibitors show significant *in vitro* activity against a broad array of non-SCLC (NSCLC) cell lines, including EGFR-mutated lines that have TKI-resistant mutations (12) and mutant KRAS cell lines (13). Thus, Hsp90 inhibitors are a promising new avenue for exploration in advanced solid tumors.

Cancer cells can develop resistance to chemotherapeutic agents, such as doxorubicin due to a variety of mechanisms, including over-expression of P-glycoprotein (14), activation of NFkB (15), and the induction of a heat shock response (16). Over-expression of Hsp90 and its co-chaperones in tumor cells results in upregulation of drug transporters, such as RLIP76 (17) resulting in resistance to chemotherapeutic agents, including doxorubicin and etoposide.

We have previously shown that the IC50 of ganetespib, a novel non-geldanamycin Hsp90 inhibitor, is 200-fold greater than 17-AAG (geldanamycin analog), in 12 different SCLC cell lines, and that ganetespib induced persistent G2/M phase arrest in SCLC cells (18). We examined the combination of ganetespib with two different topoisomerase II inhibitors, etoposide and doxorubicin (19). The combination of ganetespib and doxorubicin or etoposide significantly reduced cell viability compared to either agent alone (19). In H82-immunodeficient mice xenografts treated with ganetespib and doxorubicin, the combination of the two agents resulted in a significantly greater tumor volume reduction compared to ganetespib or doxorubicin alone. High expression of RIP1, an HSP90 client protein, contributes to apoptotic resistance through activation of the NFκB pathway (20). Doxorubicin has been shown to induce NFκB activation, rendering cells resistant to the drug. It is proposed that ganetespib could counteract the effect of doxorubicin on NFκB activation, by significantly reducing RIP1 expression. Indeed, ganetespib significantly reduced RIP1 expression in ganetespib-treated H82 and GLC4 SCLC cell lines (14).

Here we proposed that combining doxorubicin with ganetespib, a potent and novel, non-geldanamycin Hsp90 inhibitor may be able to overcome acquired drug resistance in SCLC patients.

## Materials and Methods

### Study Design

The primary objective of the study was to determine the maximum tolerated dose and establish the recommended phase II dose (RP2D) of ganetespib and doxorubicin in subjects with advanced solid tumors. The secondary objectives were to determine the dose limiting toxicities (DLTs) and to assess preliminary evidence of activity for the combination of ganetespib and doxorubicin in relapsed or refractory SCLC by determining the ORR and duration of overall response (DOR).

The dose escalation phase followed a standard 3 + 3 dose escalation scheme with 2 dose levels of ganetespib (100 and 150 mg/m^2^) administered weekly on Days 1 and 8 of a 21-day cycle, in combination with fixed dose doxorubicin at 50 mg/m^2^ on Day 1. After 4–6 cycles of the combination, continuation of single agent ganestespib was permitted in patients deriving clinical benefit. The RP2D determined at the end of the dose escalation phase was used to conduct a dose expansion study in subjects with RR-SCLC to assess if there is a signal of efficacy in this population. The treatment plan was identical to the dose escalation phase.

Treatment continued until disease progression, intercurrent illness that prevented treatment for >3 weeks, unacceptable toxicity, patient’s decision to withdraw from the study, or if study drug became unavailable. Subjects were followed for 30 days after the last dose of the study drug or death, whichever was earlier.

The trial was registered with Clinical Trials Registry (NCT02261805) and was approved by an appropriate Scientific Review Committee and an independent Institutional Review Board (IRB) prior to initiation. The study was performed in accordance with the 1964 Declaration of Helsinki and its later amendments with written informed consent obtained from all patients before study enrollment.

### Patient Selection

Key inclusion criteria were refractory solid tumors (in dose escalation phase) and RR-SCLC (in dose expansion phase), not more than three prior lines of cytotoxic therapies, age ≥18 years, ECOG performance status 0–1, adequate organ/marrow function, and life expectancy >3 months; key exclusion criteria included LVEF <50%, lifetime cumulative doxorubicin dose >150 mg/m^2^, untreated, symptomatic brain metastases, known serious cardiac illness including, but not limited to clinically significant atrial and ventricular arrhythmias and heart block, QTc >470 ms, strong inhibitors, or inducers of CYP 3A4 or 2C19 and known allergic or hypersensitivty reactions to taxanes.

### Treatment Plan

Ganetespib was administered first as a 1-h IV infusion *via* peripheral IV access at either 100 or 150 mg/m^2^ on Days 1 and 8 of each 21-day treatment cycle. After a 1-h rest period following the completion of ganetespib administration, doxorubicin was given at a dose of 50 mg/m^2^ on Day 1 of each 21-day treatment cycle *via* central venous access.

### Dose Modification Guidelines

If DLTs occurred, the protocol defined that treatment must be modified as follows: once these DLTs resolved to ≤Grade 1, the patient could resume treatment with appropriate dose reductions. For DLTs attributable to doxorubicin (myelosuppression, mucosal ulceration, injection site reactions, cardiotoxicity), the dose of doxorubicin was reduced to dose level 1 (40 mg/m^2^). For all other DLTs, the dose of ganetespib was reduced by one dose level (80 mg/m^2^). Occurrence of DLTs in the first cycle at the first dose level and re-occurrence of DLTs in the first cycle following one dose reduction in any single patient was defined as cause for termination of treatment for that patient.

Treatment cycles followed the 3-week cycle length. The start of a new cycle could be delayed up to 3 weeks. If treatment was held for >3 weeks from the time the last dose was due, then the subject would be removed from the study. Ganetespib treatment on Days 8 and 15 would be omitted rather than delayed, as per detailed guidelines noted in the protocol.

### Safety, Pharmacokinetic, and Efficacy Assessments

#### Dose Limiting Toxicities

Any Grade 4 hematologic toxicity or any Grade 3 or higher non-hematologic toxicity except nausea, vomiting, diarrhea, alopecia, or >Grade 3 diarrhea, nausea or vomiting that lasts longer than 72 h despite maximal medical therapy, and hypersensitivity reactions >Grade 3 with pre-medication were defined as DLTs.

Treatment emergent adverse events (TEAEs) were assessed from the time of study drug administration until 30 days following discontinuation of study drug according to the National Cancer Institute Common Toxicity Criteria for Adverse Events (AEs) version 4.0.

Adverse events and serious AEs were reported to study sponsor and the Georgetown University IRB. In addition, data safety was monitored on a quarterly basis by an internal data and safety monitoring committee.

Screening assessments included history and physical exam, including vital signs, ECOG performance status, 12 lead electrocardiogram (EKG), MUGA scan, or 2D echocardiogram to assess left ventricular (LV) function, complete blood count, and comprehensive metabolic panel including electrolytes, and serum beta-HCG in women of child bearing age. On-study assessments included history and physical exam, including vital signs, ECOG performance status, 12 lead EKG, complete blood counts, and comprehensive metabolic panel, including electrolytes on Day 1 of each cycle; in addition, EKG was also repeated prior to each ganetespib infusion and at 24 h post-ganetespib in cycles 1, 2, and 3. Tumor assessments/radiologic evaluations were performed every 6 weeks ± 7 days. End of study assessment included a MUGA scan or 2D echocardiogram to evaluate LV function.

#### Statistical Analyses

The sample size was based on the dose escalating probabilities calculated using the standard 3 + 3 dose escalation scheme. If the true DLT rate at a given dose is 10, 20, 30, 40, 50, or 60%, then the probability of dose escalation is 0.91, 0.71, 0.49, 0.31, 0.17 and 0.08, respectively. No specific statistical hypothesis tests were planned, with descriptive statistics used for the patient demographic information, analysis of safety events, and tumor response data. The study population for all safety analyses included patients who received ≥1 dose of study medication. The study population for all preliminary efficacy analyses included patients who had received at least 2 cycles of therapy. Once the RP2D was established, enrollment of 10 patients with SCLC was planned in the dose expansion phase at the RP2D. The sample size of 10 additional patients treated at the RP2D was chosen to ensure a reasonably high chance of identification of toxicities that will occur in at least 14% of patients. That is, with a total of 16 patients treated at the RP2D, there would be a 90% chance that at least one patient will experience a specific toxicity, if the true probability of that toxicity is 0.134 or greater.

Exploratory efficacy endpoints included ORR and DOR. ORR included confirmed complete response (CR) and partial response (PR) and was based on Response Evaluation Criteria in Solid Tumors version 1.1. ORR was calculated for all patients with ≥1 measurable lesion at baseline. Time to progression (TTP) was defined as the number of days from start of treatment to disease progression. DOR was defined as the number of days from the day criteria were met for CR or PR (whichever was recorded first) to the date that PD was objectively documented. If a patient was still responding, the patient’s data were censored at the date of the last study visit at which timepoint a tumor assessment was performed. If a patient never experienced a CR or PR, the patient’s data were censored on the day of the first dose. Analyses of change and/or percent change from baseline for tumor size were performed for each scheduled post-baseline visit and the final visit.

## Results

A total of 11 subjects were enrolled in the study from November 2014 to January 2016: 9 were enrolled in the phase I dose escalation portion of the study (3 subjects at dose level 1 and 6 subjects at dose level 2) and the RP2D was 150 mg/m^2^ of ganetespib and 50 mg/m^2^ of doxorubicin. The maximum tolerated dose was not reached. Two additional patients with relapsed/refractory SCLC were enrolled on the phase II dose expansion portion of the study. The study was terminated prematurely by the sponsor who ceased development of ganetespib.

Baseline demographics and patient characteristics are depicted in Table 1.

**Table 1:** Baseline demographics and patient characteristics.

Demographic	Value
Sex distribution	
Male	7
Female	4
Race	
Caucasian	7
Black	3
Asian	1
Median age in years (range)	59 (36–76)
Performance status	
ECOG PS 1	8
ECOG PS 0	3
Primary tumor site	
Non-small cell lung cancer (SCLC)	4
SCLC	5
Mesothelioma	1
Pancreatic neuroendocrine carcinoma	1
Prior therapies	
One prior therapy	6
Two prior therapies	2
Three prior therapies	3

All 11 subjects completed at least 1 cycle of therapy and were included in the analyses of safety outcomes. Three subjects did not complete at least 2 cycles of study therapy and were excluded from any preliminary efficacy analyses.

### Safety Outcomes

The most common grade 1/2 AEs were diarrhea, nausea/vomiting, fatigue, and AST or ALT elevation. There were three grade 3 AEs (one each of ALT elevation, anemia, and neutropenia) and one grade 4 AE of neutropenia that did not meet criteria for DLT. No DLTs were observed during the dose escalation phase of the study. A complete listing of TEAEs is provided in Table 2.

**Table 2:** Treatment emergent adverse events.

Adverse event	Grade 1	Grade 2	Grade 3	Grade 4
Diarrhea	8	3	0	0
Nausea/vomiting	9	0	0	0
Fatigue	4	2	1	0
Anorexia	1	1	0	0
Mucositis	0	1	0	0
AST or ALT elevation	5	0	1	0
Anemia	1	2	1	0
Leukopenia	2	1	0	0
Neutropenia	1	2	1	1
Thrombocytopenia	2	0	0	0
Hypomagnesemia	3	0	0	0
Hypophosphatemia	1	1	0	0
Hypokalemia	2	1	0	0
Infusion reactions	0	1	0	0

### Efficacy Outcomes

All but two patients came off the study due to disease progression; one patient came off the study due to worsening fatigue and another patient came off the study due to study termination. Three patients did not complete at least two cycles of study therapy and were excluded from the analyses of efficacy outcomes. No patient achieved a CR. Two of eight patients achieved a PR (ORR of 25%) and both were patients with platinum-sensitive relapsed SCLC. Of the three other patients with SCLC, all of whom had platinum-refractory disease, one had progressive disease as best response after two cycles, and the other two patients had best response of stable disease lasting 18 and 25 weeks. The DOR was 207 days for one patient in the study and 188 days for the other patient (whose study treatment was prematurely terminated after 6 cycles due to study closure). The median duration of response was 197.5 days (range, 207–188). All other subjects achieved stable disease as the best response. The median TTP was 137 days (range, 29–242) for the intent-to-treat population.

## Discussion

Chemotherapeutic resistance in SCLC remains a challenging area of research. Understanding the mechanistic underpinnings of chemoresistance is the key to overcoming such resistance. Our study exploited the dependence of doxorubicin resistance on the induction of the heat shock response by utilization of a novel non-geldanamycin Hsp90 inhibitor, viz. ganetespib. In summary, the findings of this phase Ib/II study demonstrate that the RP2D of ganetespib in patients with advanced solid tumors is 150 mg/m^2^ given weekly on Days 1 and 8 with doxorubicin given at 50 mg/m^2^ on day 1 in 21-day cycles. Ganetespib in combination with doxorubicin was generally well tolerated and the maximum tolerated dose was not reached based on the study design. The types and incidences of TEAEs were consistent with the known side effects of ganetespib from prior studies reported in the investigators’ brochure and included diarrhea, nausea, fatigue, and transaminitis. No ophthalmic or unexpected cardiac side effects, including clinically significant QT prolongation or cardiac arrhythmias were noted, as expected for this subclass of second generation Hsp90 inhibitors, unlike the first generation Hsp90 inhibitors with a benzoquinone moiety, such as 17AAG (Tanespimycin) and 17DMAG (Alvespimycin). There was preliminary evidence of antitumor activity, with the combination of ganetespib and doxorubicin yielding an ORR of 25%. Patients with SCLC experienced the greatest antitumor activity, with an ORR of 40% (2 of 5 subjects with PR).

In conclusion, this phase Ib/II dose escalation study demonstrates that ganetespib and doxorubicin can be safely combined in patients with advanced solid tumors with a tolerable safety profile. Preliminary data show antitumor activity in advanced solid tumors, with clinically meaningful benefit in SCLC. The findings reported herein support further evaluation of ganetespib in combination with doxorubicin in this disease. It is interesting to note that single agent ganetespib was tested in SCLC in a single institution study and no objective responses were noted in this study (Gandhi et al. Personal Communication). A phase III randomized controlled trial of docetaxel alone or in combination with ganetespib in advanced NSCLC (GALAXY-2) showed no improvement in progression-free survival or overall survival, including pre-specified populations of patients with advanced disease diagnosis more than 6 months prior to study enrollment and with elevated lactate dehydrogenase (21). These results likely influenced the sponsor’s strategic decision to halt further development of ganetespib. However, our study demonstrates that there is room for further investigation of similar second-generation Hsp90 inhibitors in the arena of SCLC and other highly aggressive neuroendocrine malignancies. It may be important to identify appropriate predictive biomarkers for clinical trials of Hsp90 inhibition in order to maximize efficacy and minimize subject exposure to toxicities. The second-generation Hsp90 inhibitors are small molecules encompassing the resorcinol moiety of radiciol or are purine derivatives, and include compounds, such as NYP-AUY922, AT-13387, and KW-2478 (22). Recently, everolimus, a small molecule mTOR inhibitor, has been approved in the treatment of non-functional metastatic neuroendocrine tumors of the lung or gastrointestinal tract based on the progression-free survival benefit seen in the RADIANT-4 trial (23). It has been shown that Hsp90 inhibition may subsequently trigger the development of a heat shock response by upregulation of Hsp70, and the latter can be blocked by pre-treatment with mTOR inhibitors, such as cycloheximide in multiple tumor types (24). Therefore, it may be worth exploring combinations of second-generation Hsp90 inhibitors with mTOR inhibitors like everolimus.

## Author Contributions

Substantial contributions to the conception or design of the work: GG, DS. Acquisition, analysis, or interpretation of data for the work: GG, DS, SL, JC, JT, JK, and HW. Drafting the work or revising it critically for important intellectual content: GG, DS, SL, and HW. Final approval of the version to be published: GG, DS, SL, JC, JT, JK, and HW. Agreement to be accountable for all aspects of the work in ensuring that questions related to the accuracy or integrity of any part of the work are appropriately investigated and resolved: GG, DS, SL, JC, JT, JK, and HW.

## Conflict of Interest Statement

The authors declare that the research was conducted in the absence of any commercial or financial relationships that could be construed as a potential conflict of interest.
